# Solid tumors of the pancreas can put on a mask through cystic change

**DOI:** 10.1186/1477-7819-9-79

**Published:** 2011-07-19

**Authors:** Kwang Yeol Paik, Seong Ho Choi, Jin Seok Heo, Dong Wook Choi

**Affiliations:** 1 Department of Surgery, The Catholic University of Korea, Yeouido St.Mary's Hospital, Seoul, Korea; 2 Department of Surgery, Samsung Medical Center, Sungkyunkwan University School of Medicine, Seoul, Korea

**Keywords:** solid, cystic, pancreas, tumor

## Abstract

**Background:**

Solid pancreatic tumors such as pancreatic ductal adenocarcinoma (PDAC), solid pseudopapillary tumor (SPT), and pancreatic endocrine tumor (PET) may occasionally manifest as cystic lesions. In this study, we have put together our accumulated experience with cystic manifestations of various solid tumors of the pancreas.

**Methods:**

From 2000 to 2006, 376 patients with pancreatic solid tumor resections were reviewed. Ten (2.66%) of these tumors appeared on radiological imaging studies as cystic lesions. We performed a retrospective review of medical records and pathologic findings of these 10 cases.

**Results:**

Of the ten cases in which solid tumors of the pancreas manifested as cystic lesions, six were PDAC with cystic degeneration, two were SPT undergone complete cystic change, one was cystic PET, and one was a cystic schwannoma. The mean tumor size of the cystic portion in PDAC was 7.3 cm, and three patients were diagnosed as 'pseudocyst' with or without cancer. Two SPT were found incidentally in young women and were diagnosed as other cystic neoplasms. One cystic endocrine tumor was preoperatively suspected as intraductal papillary mucinous neoplasm or mucinous cystic neoplasm.

**Conclusions:**

Cystic changes of pancreas solid tumors are extremely rare. However, the possibility of cystic manifestation of pancreas solid tumors should be kept in mind.

## Background

Pancreatic cystic tumors are frequently and increasingly diagnosed due to improvement of imaging quality and increased frequency of imaging diagnosis. Interestingly, solid pancreas neoplasma may undergo degeneration or change in its structure to appear as cystic tumors, masking its originality as a solid pancreas neoplasm. Clinically, most of pancreatic cystic tumors are benign, but cystic degeneration of solid tumors are frequently malignant, especially pancreatic ductal adenocarcinoma (PDAC). As the significance of the cystic lesions emerged, cystic forms of otherwise typically solid tumors were also better characterized [1]. Solid-pseudopapillary tumor (SPT) and PDAC may exhibit large cystic degenerations with hemorrhagic and necrotic debris on rare occasions [2]. Such cystic tumors are often mistaken for pseudocyst of the pancreas by imaging studies and macroscopic examinations [3]. Other forms of pancreatic cystic lesions, for example cystic pancreatic endocrine tumor (PET), are extremely uncommon. We report solid pancreatic tumors exhibited as cystic tumors in imaging or gross appearances before pathologic examination in a single referred institute.

## Methods

From 2000 to 2006, 376 patients at our center who underwent pancreas solid tumor resection (PDAC, PET, etc.) and patients who were diagnosed with SPT were reviewed retrospectively. Ten (2.66%) of these 376 tumors were diagnosed on radiological imaging as cystic lesions. Any tumors with the impression of solid or mixed cystic component on image findings were not included in this study. Medical records and pathologic findings were reviewed retrospectively. Solid pancreatic tumors include PDAC, PET, gastrointestinal stromal tumor (GIST), metastatic tumor, and schwannoma. We excluded cystic pancreas tumors such as serous cystic neoplasm (SCN), mucinous cystic neoplasm (MCN), intraductal papillary mucinous neoplasm (IPMN) in this study. SPT is well known as a tumor with cystic manifestation and can contain mixed solid and cystic portions. We found six SPT cases displaying cystic changes, and hence were included in this study. But we excluded four SPT cases containing calcification in the wall or any small solid components found on radiologic or pathologic report.

## Results

Cases of six PDAC with cystic degenerations, two cystic changes of the SPT, one cystic PET, and one cystic schwannoma were included in this study.

### PDAC

The mean age of patients with PDAC cystic changes were 62.7 years (38-78 years). All six patients had abdominal or flank pain and one showed jaundice. CT images were reviewed but endoscopic ultrasonogram (EUS) or Positron Emission Tomogaraphy images were not performed. Of the six cases, five showed elevated carbohydrate antigen (CA 19-9) levels (59-2077 IU/ml) where as one showed normal CA 19-9 level. Initially based on clinical manifestation and imaging study findings, three were diagnosed as 'pseudocyst' with or without PDAC and three lesions were suspected malignant IPMN or other form of cystic neoplasms. Two patients with suspected 'pseudocyst with cancer' had history of chronic pancreatitis. However, one other patient with suspected 'pseudocyst' in imaging findings did not have history of pancreatitis. This patient showed the largest cyst and was referred to our center after external drainage of the cyst. CA19-9 level of the cystic fluid was 24000 IU/ml but no malignant cells were found. However, we decided to proceed with operation of the pancreatic cyst due to sustained pain after external drainage and also because we concluded that the possibility of hidden malignancy could not be completely ruled out. We did not perform routine cystic fluid aspiration or tumor marker tests.

**Figure 1:**
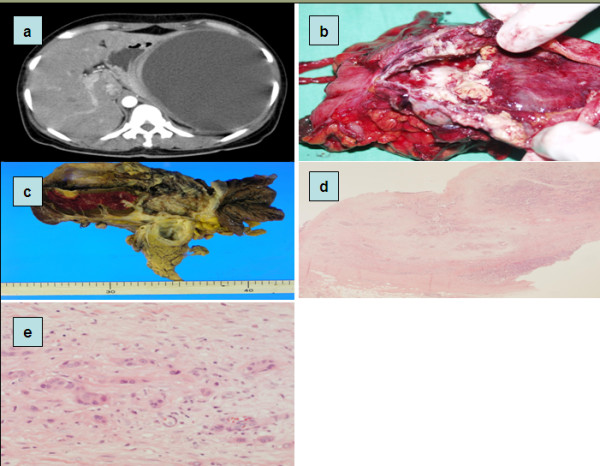
**PDAC with cystic degeneration**. (a) CT shows huge cystic pancreas mass in tail portion. (b,c) Macroscopic appearance of the tumor showing big degenerative cyst which wall is severly thickened. (d,e) Microscopic finding of tumor wall contains cancer cell with poorly differentiation.

Of the PDAC tumors, the mean size of tumors was 7.3 cm (3.0-11.0 cm). Two patients with cystic PDAC showed multiple lesions in the pancreas. We performed two pancreaticoduodenectomies, three distal pancreatectomies, and one total pancreatectomy. All tumors except one which was suspicious of IPMN had grossly detectable invasion to adjacent organ such as the colon, stomach, and kidney. Hence four patients underwent transverse colectomy for severe adherence and combined gastrectomy, and one patient underwent adrenalectomy. Pathologic findings revealed invasion to adjacent organs in three lesions, showing cancer cell invasion of the cystic wall (Figure 1). The clinical features of all six patients with PDAC cystic degeneration are summarized in Table 1.

**Table 1:** Clinical aspects in patient with the pancreatic ductal adenocarcinoma cystic degeneration.

Age (mean,range) (yr)	62.7	
Gender (male/female)		2/4
Chief complaint	Abdominal pain	5
	jaundice	1
CA 19-9	< 37 IU/ml	2
	≥ 37 IU/ml	4 (range: 59~2077)
Radiologic diagnosis	pseudocyst	3
	Cystic neoplasm	3
Location	head	2
	body and tail	4
Operation	pancreaticoduodenectomy	2 (add colectomy 1)
	distal pancreatectomy	3 (add colectomy 2)
	total pancreatectomy	1 (add gastrectomy and colectomy 1)
Size (mean,range) (cm)	7.3 (3.0 ~ 11.0)	

(n = 6)

### SPT

**Figure 2:**
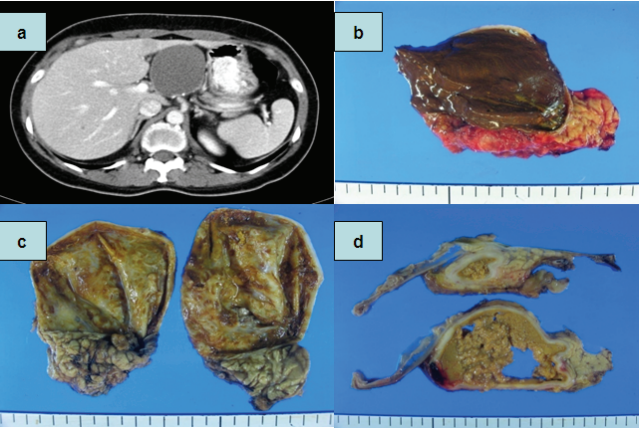
**Solid pseudopapillary tumor with cystic degeneration**. (a) CT shows cystic mass in pancreas head portion. (b-d) Gross appearance with cyst contains chocolate like materials suggestive with necrotic debrids.

Two cystic SPTs were detected incidentally in two young women by screening. Based on imaging findings, diagnosis of MCN were made for both patients and in one lesion, dermoid cyst or hemorhhagic cyst was suspected. Although focal calcifications were found in cystic walls of both, SPT was not suspected. Each tumor was located in the pancreas head and tail portion, and they underwent Pylorus preserving pancreaticoduodenectomy (PPPD) and distal pancreatectomy (DP) respectively. Both tumors contained muddy chocolate materials which is suspicious of hemorrhagic debris (Figure 2).

Immunohistochemistry test was performed on the cells of the cyst walls and the results showed positive findings for CD10 but negative findings for chromogranin and synaptophysin based upon which the pathological diagnosis of SPT was made, with one revealing benign features whereas the other showed low grade malignancy. Clinical features of the SPT tumors are depicted in Table 2.

**Table 2:** Two cases of the solid pseudopapillary tumor with cystic degeneration.

	Case 1 (F/34)	Case 2 (F/37)
Chief complaint	incidental	incidental
Lab	normal	normal
Radiologic diagnosis	Dermoid cyst, hemorrhagic cyst, MCN	MCN
Operation	Distal pancreatectomy	PPPD
Pathology	benign	Cystic degeneration with necrosis, low grade malignancy
Size	9 × 8 cm	8 × 7 cm
Immunohistochemistry	CD 10 (+) Chromogranin (-) Synaptophysin (-)	CD 10 (+)

MCN: mucinous cystic neoplasm

PPPD: pylorus preserving pancreaticoduodenectomy

CD: cluster of differentiation

### Cystic PET

A cystic mass on the pancreas head suspected of MCM or IPMN was found in a computed tomography (CT) performed on a 53 year old female patient which was done for the purpose of evaluating uncontrolled DM and weight loss. (Figure 2a) She underwent PPPD and the mass found was measured to be 3.5 × 3.3 cm. This tumor displayed CD10 negative, chromogranin positive, synaptophysin positive, and vimentin weak positive findings on immunohistochemistry which led to a pathological diagnosis of well differentiated benign cystic PET.

### Cystic schwannoma

CT findings of a 77 year old female patient with epigastric pain revealed a cystic tumor of the pancreas head which contained a papillary protruding solid mass within the cyst and a diagnosis of either SPT or PET was made based upon these findings. PPPD was performed and the tumor was found to be filled with serous fluid, which was stained positive for S100 protein. Thus, the tumor was confirmed as a cystic schwannoma.

The clinical features of the ten patients reviewed in this study are summarized in Table 3.

**Table 3:** Summary of pancreas solid neoplasms with cystic manifestation.

	diagnosis	Age/Sex	Preoperative Dx	operation
1	PDAC	78/M	Pseudocyst or PDAC	DP/Lt.adrenalectomy/colectomy
2	PDAC	57/M	Acute pancreatitis with pseudocyst/PDAC	TP/TG/colectomy
3	PDAC	53/F	Malignant IPMN	PPPD
4	PDAC	79/F	Cystic neoplasm	PD/colectomy
5	PDAC	71/F	MCN	DP
6	PDAC	38/F	pseudocyst	DP/colectomy
7	SPT	34/F	Cystic neoplasm	DP
8	SPT	37/F	MCN	PPPD
9	PET	55/F	MCN or IPMN	PPPD
10	schwannoma	73/F	SPT	PPPD

PDAC: pancreatic ductal adenocarcinoma

DP: distal pancreatectomy

TP: total pancreatectomy

TG: total gastrectomy

IPMN: intraductal papillary mucinous neoplasm

MCN: mucinous cystic neoplasm

SPT: solid pseudopapillary tumor

PPPD: pylorus preserving pancreaticoduodenectomy

PET: pancreatic endocrine tumor

PD: pancreaticoduodenectomy

## Discussion

Owing to recent improvement in abdominal imaging and invasive diagnostic techniques, an increasing number of pancreas cystic lesions are identified in patients who are clinically indolent or silent. In addition to the well-known pancreas cystic lesions, the differential diagnosis of pancreatic cysts also includes cystic changes in otherwise typically solid tumors of this organ [4]. It is important to recognize this group, because unlike well-known pancreas cystic lesions, these are often low grade malignancies as in the case of SPT or true carcinomas as in the case of cystic changes in ductal adenocarcinoma [1]. Cystic feature of solid tumor of pancreas may result due to necrosis, hemorrhage and degeneration of tumor cells. Adsay [1] described these cystic categories in detail.

PDAC with cystic changes have been reported in some cases [3,5-7]. The largest single institute series of cystic PDAC was reported in Germany, in which thirty (7.2%) of 418 cystic tumors of the pancreas were PDAC presenting cystic features [8]. These lesions could be misdiagnosed as pseudocysts based upon imaging studies before operation. Half of our cases of PDAC with cystic change were originally diagnosed as pseudocysts with or without cancer before operation. Cystic epithelial cell linings were absent in our cases. German cases of cystic PDAC showed same staining patterns as the PDAC [8]. Central necrosis may result in a unilocular cyst surrounded by a rim of viable malignant tissue [9]. Pseudocysts in patients with no history of chronic pancreatitis should be closely evaluated for differential diagnosis of malignancy [3,10,11]. In cases without chronic pancreatitis, it is possible for pseudocysts accompanying PDAC to develop due to obstruction of the pancreatic duct by the tumor [10]. Proper sampling of pseudocysts is essential and these samples should consist of cyst walls obtained during open procedures or cyst contents obtained during minimal access drainage procedures [8]. Kosmahl et al [9] suggested that the discrepancy between findings of his series in which PDAC with cystic features are frequent and other studies in which these findings are close to nonexistent may be explained by the assumption that cystic features in PDAC have not attracted much attention and have, therefore, probably been neglected. During a period of six years, our cases showed a frequency of 1.6% of PDAC with cystic features. Probably cystic PDAC are more occupied in cystic pancreas tumor due to large number of observational small size pancreatic tumors waiting surgical option in clinical fields. In fact, Kosmahl [9] classified cystic PDAC as neoplastic epithelial type of cystic pancreas neoplasm and lesion in 2004. In Korea, one patient with PDAC coexisting with pancreatitis and pseudocyst was reported [12].

In our series, cystic PDAC showed aggressive behavior in CT findings which was checked before operation. Cyst wall abutted adjacent organs such as the transeverse colon, stomach, and kidney. Four patients underwent combined organ resection. If the pancreas cystic mass shows aggressive shape on imaging studies, malignancies such as PDAC should be suspected. We made operation decisions based upon CT as the only imaging modality. If we performed Positron Emission Tomogaraphy (PET) in these cases, malignancy would have been easily suspected and these cases would have been prepared for more adequate therapy. Elevated CA 19-9 may also be another clue of malignancy, especially when pseudocyst is suspected on image findings.

SPT can show degeneration with cystic features. They usually start as solid tumors and undergo massive degeneration giving rise to cystic appearances on radiological imaging [13]. It is now known that the cavities that are formed in SPTs are not 'true' cysts (there is no epithelial lining) but rather represent a necrotic/degenerative process in which the cystic areas consist of blood, necrotic debris and foamy macrophages [1]. In our two cases, MCN was suspected preoperatively. SPT with cystic change is very rare and no single center report have existed. Recently, CD10 expression and APC/ß-catenin pathway and cyclin-D1 alterations were found to be almost uniformly present (> 90%) in SPTs. This interesting finding is very helpful diagnostically, and may prove to be important in unraveling the pathogenesis of this peculiar tumor [1]. We diagnosed this cystic SPT on basis of CD10 positive findings.

Recently, Bordeianou et al reported the largest series of cystic PET. PET is no longer considered a pure solid neoplasm, as it frequently appears with cystic manifestations. They suggested that cystic PET are more common than previously thought, and that it should be included in the differential diagnosis of cystic pancreas neoplasms [14]. It has been assumed that cystic PET are similar to solid PET as far as behavior and malignant potential [15,16]. This assumption derives from the hypothesis that cystic PET arise as a result of tumor necrosis within solid PET [17]. Cystic PET are larger and more likely to be symptomatic than solid PET [14,16]. Our case of patients with cystic PET had no clinical symptoms and had a borderline size of 3.5 cm. The tumor contained serous fluid and pathological diagnosis of cystic PET was made according to immunohistochemistry findings which showed chromogranin, synaptophysin positive findings and CD10 negative findings. Cystic schwannoma was very rare compared to previously documented pancreas cystic neoplasms. Few case reports were published [18,19]. Including our cases, all cystic schwannomas stained positive for S100. In our series, consecutive ten cases of cystic features of solid pancreas neoplasm were collected retrospectively, and diagnosis was made depending upon pathologic review. Cystic pancreatic neoplasm can hide its originality of being a solid neoplasm with cystic changes. We always make effort to make differential diagnosis of pancreatic cystic neoplasms using clinical and pathological diagnostic tools available. The clinically small size of pancreatic cystic neoplasms can conceal malignant potentials especially of its solid counterpart.

## Conclusions

Cystic formations of the pancreatic solid tumors are rare. However, the possibility of cystic manifestation within pancreas solid tumors should be kept in mind.

## Competing interests

The authors declare that they have no competing interests.

## Authors' contributions

All authors contributed to treatment of patients, collection of data, review of results and manuscript, and approval of the final draft.
